# Chemically-Induced Production of Anti-Inflammatory Molecules in Microalgae

**DOI:** 10.3390/md16120478

**Published:** 2018-11-30

**Authors:** Zaida Montero-Lobato, María Vázquez, Francisco Navarro, Juan Luis Fuentes, Elisabeth Bermejo, Inés Garbayo, Carlos Vílchez, María Cuaresma

**Affiliations:** 1 Algal Biotechnology Group, CIDERTA, RENSMA and Faculty of Sciences, University of Huelva, 21007 Huelva, Spain; mariazaida.montero@alu.uhu.es (Z.M.-L.); maria.vazquez@ciecema.uhu.es (M.V.); jlfuentes@dqcm.uhu.es (J.L.F.); elisabeth.bermejo@dqcm.uhu.es (E.B.); garbayo@uhu.es (I.G.); maria.cuaresma@dqcm.uhu.es (M.C.); 2 Department of Integrated Sciences, Cell Biology, Faculty of Experimental Sciences, University of Huelva, 21007 Huelva, Spain; fnavarro@dbasp.uhu.es

**Keywords:** anti-inflammatory, bioactive molecules, microalgae, polysaccharides, carotenoids, polyunsaturated fatty acids

## Abstract

Microalgae have been widely recognized as a valuable source of natural, bioactive molecules that can benefit human health. Some molecules of commercial value synthesized by the microalgal metabolism have been proven to display anti-inflammatory activity, including the carotenoids lutein and astaxanthin, the fatty acids EPA (eicosapentaenoic acid) and DHA (docosahexaenoic acid), and sulphated polysaccharides. These molecules can accumulate to a certain extent in a diversity of microalgae species. A production process could become commercially feasible if the productivity is high and the overall production process costs are minimized. The productivity of anti-inflammatory molecules depends on each algal species and the cultivation conditions, the latter being mostly related to nutrient starvation and/or extremes of temperature and/or light intensity. Furthermore, novel bioprocess tools have been reported which might improve the biosynthesis yields and productivity of those target molecules and reduce production costs simultaneously. Such novel tools include the use of chemical triggers or enhancers to improve algal growth and/or accumulation of bioactive molecules, the algal growth in foam and the surfactant-mediated extraction of valuable compounds. Taken together, the recent findings suggest that the combined use of novel bioprocess strategies could improve the technical efficiency and commercial feasibility of valuable microalgal bioproducts production, particularly anti-inflammatory compounds, in large scale processes.

## 1. Microalgae as Source of Anti-Inflammatory Compounds

In recent years, the pharmacy of the sea has become a new paradigm for the discovery and development of novel drugs and bioactive compounds. The increasing need for getting drugs with no or minimal toxic side effects is one of the main reasons that motivates the search for bioactive compounds from natural sources, including anti-inflammatory active molecules from microalgae.

Much has been written about the outstanding metabolic properties of microalgae that make them particularly attractive as a natural source for bioactive molecules production [1,2,3]. Regarding the topic of this review, a variety of microalgal metabolites which display anti-inflammatory activity can accumulate in the cells to a certain extent. The diversity in chemical nature, structures and biosynthesis pathways of the most abundant anti-inflammatory molecules synthesized by microalgae are well known and have been many times reported in several noticeable reviews [4,5,6]. Among them, some carotenoids, PUFA (polyunsaturated fatty acids) and carbohydrates displaying anti-inflammatory activity have been reported [7] which also comply with two requisites a target valuable product to be produced by microalgae or cyanobacteria should meet: (i) Being accumulated at relatively high concentrations in microalgal cells grown under standard cultivation conditions, and (ii) being over produced as an algal response to suboptimal cultivation conditions or when they are subjected to chemical and/or physical stress. These suboptimal or stress conditions can be related to changes in nutrient concentration, changes in physicochemical parameters, including pH, temperature, light quality and irradiance, or addition of chemicals triggering the overproduction of target molecules [3,8].

Table 1 summarizes some of the most marketed microalgae species used for accumulation of carotenoids, PUFA and carbohydrates, biochemical molecules groups that include the main anti-inflammatory microalgal compounds.

It is apparent that the proven anti-inflammatory activity of the biomolecules listed in Table 1 increases the value of the algal biomass as a source of functional components for the production of food supplements and novel foods or the production of natural ingredient-based health products. Before proven active against inflammation, some of the most valuable microalgal molecules, listed in Table 1, were reported to be commercially valuable due to their antioxidant properties which confer on the microalgal biomass high potential as a nutritional supplement. For instance, this is the case for the carotenoids astaxanthin (3,3′-dihydroxy-β,β-carotene-4,4′-dione), lutein (3R,3′R,6′R-βε-carotene-3,3′-diol) and β-carotene (β,β-carotene), which are considered added-value molecules produced by microalgae traditionally demanded by the nutrition market for the production of food supplements [15,16]. The role of the aforementioned microalgal molecules in inflammation and chronic inflammation has been extensively described in a specific review [7], and other related papers [15,17]. Besides the anti-inflammatory activity, the antioxidant capacity displayed by many of these natural molecules increases their value as natural products with potential benefits to human health [18,19,20]. In addition to those molecules listed in Table 1, phycobiliproteins, phenolic compounds and several carotenoid-isomers are also among those microalgal compounds displaying anti-inflammatory activities and being potentially valuable for commercial applications related to human health care [7,21].

One of the main advantages of microalgae when used for the production of valuable compounds in large scale production systems is the high areal biomass productivities. The systematic production of microalgae biomass at large scale in addition to the high productivity of microalgal cultures under well established and controlled cultivation conditions [22] make them valuable as a natural source for the production of a range of bioactive compounds, including molecules displaying anti-inflammatory activity [3]. The low generation times of microalgal growth and the intensive control of cultivation parameters in photobioreactors make the production of bioactive compounds-enriched microalgal biomass technically and economically feasible at large scale throughout the year at suitable latitudes. Moreover, the algal metabolic plasticity and rapid response to changes in physicochemical conditions ease to address the accumulation of specific metabolic products in large-scale production processes. In this review, the specific microalgae species and cultivation strategies that could be more suitable to enhance accumulation of anti-inflammatory microalgal molecules are discussed.

Only a few microalgae species are currently being produced with a commercial purpose in spite of the huge diversity existing in nature which according to literature accounts for about 300,000 species, out of which only 10% (30,000) have been documented [23]. Thus, most of the existing microalgae species are still to be discovered and tested for biotechnological exploitation. The worldwide microalgal market for food, feed or high-value ingredients production is mostly composed of a limited number of algal species belonging to the following genera: *Chlorella*, *Spirulina* (*Arthrospira*), *Dunaliella*, *Haematococcus*, *Nannochloropsis*, *Scenedesmus*, *Isochrysis*, *Porphyridium* and *Phaeodactylum* [16]. Until 2014, only a few high-value molecules produced with microalgae had reached the food and feed market: (i) Several pigments, such as the carotenoids β-carotene and astaxanthin, and the protein complex phycocyanin; and (ii) polyunsaturated fatty acids, such as EPA (eicosapentaenoic acid) and DHA (docosahexaenoic acid). All these compounds have been found to display anti-inflammatory activity, though most of them reached the market thanks to properties other than anti-inflammation. In addition, a peptide from *Phaeodactylum tricornutum* reached the market based on its anti-inflammatory properties only [16]. Thus, there is still a big room for discovering microalgae species with the capacity to accumulate anti-inflammatory compounds that could reach the market.

With no doubt genetic engineering techniques currently offer a large number of procedures to obtain modified strains which are designed to display specific functionalities. However, several factors still make the use of genetically modified microalgae difficult at commercial production scale; for instance, a still little positive consumer perception towards genetically modified organisms and the restrictive regulations on genetically modified organisms in many countries slow down development of industrial production of microalgae enriched in high-value compounds. However, the huge diversity of wild microalgae species that remain unexplored and unexploited should still for a long time allow addressing research on their natural potential for the production of target molecules by means of triggering key biosynthetic pathways through the use of specific chemicals and cultivation conditions [8,23]. This is one of the key messages of this review article, and the information and discussion below directly focus on this approach.

The biotechnological potential of most marketed microalgae species is actually very well known, and the expectations to unveil novel, abundant bioactive compounds from non-extremophilic microalgae are decreasing. In coming years, the production of novel microalgal compounds should expectedly be carried out from novel microalgal species isolated from locations where they could eventually be further produced at large scale. In this respect, extremophilic microalgae are microorganisms with unique metabolic capabilities yet unexploited, with a competitive advantage (as compared to non-extremophiles) to grow in open systems under restrictive cultivation conditions (for example, highly acid pH or very low temperature) which limit microbial contamination. For instance, *Dunaliella salina* is an outstanding example of extremophilic microalga commercially used for the production of a high value compound which displays anti-inflammatory activity, β-carotene [24].

## 2. Chemically-Induced Oxidative Stress to Improve Production of Anti-Inflammatory Compounds

### 2.1. Chemicals Triggering Accumulation of Anti-Inflammatory Compounds

Marketing of anti-inflammatory compounds obtained from microalgae is possible only if commercial feasibility of the product production process is achieved. A production process could become commercially feasible if the biosynthesis yields and productivity of target molecules are high and the overall production process costs are minimized. The productivity of anti-inflammatory molecules depends on each microalgal species and the specific cultivation conditions that boost the biosynthesis pathways involved. These conditions must be optimized for each microalgal species.

A number of anti-inflammatory molecules obtained from microalgae have also been shown to display high antioxidant capacity, thus they could in theory be produced under oxidative stress conditions. A list of the most abundant microalgal molecules with both anti-inflammatory and antioxidant activities should include the pigments β-carotene [25], astaxanthin [26], lutein [27], zeaxanthin [28] and phycobiliproteins [29]. The carotenoids exhibit high antioxidant activity which has been reported to positively impact human health, based on the chemical ability of carotenoids to scavenge reactive oxygen species (ROS) produced in the cell by the oxidative metabolism [7,15,30].

In addition to the aforementioned antioxidant pigments, LC-PUFAs (long chain polyunsaturated fatty acids, including EPA and DHA) have also been proven to exert antioxidant activity, which for instance was exemplified by studies in human vascular endothelial cells demonstrating reduced excretion of lipid peroxidation products after omega 3-intake and superoxide scavenging by LC-PUFAs [31]. The third group of anti-inflammatory molecules produced by microalgae, the polysaccharides, has also been proven to exert antioxidant activity, and their applications and benefits to human health can be found in several outstanding reviews published in recent years [7,32]. Polysaccharides isolated from *Porphyridium* [33] and *Rhodella* [34] are noticeable examples of antioxidant microalgae polysaccharides.

In addition to the above mentioned compounds, a number of other microalgal molecules have recently been reported which also have high antioxidant capacity besides anti-inflammatory activity: Phenolic compounds (flavonoids) [21], peptides [35], and the carotene isomers trans-β-carotene and 9-cis-β-carotene.

According to the aforementioned antioxidant capacity of a large number of microalgal molecules which also display anti-inflammatory activity, some of the main chemical strategies to enhance their biosynthesis and intracellular accumulation could be based on inducing oxidative stress in the cells which might result in increased production rates of antioxidant molecules. In this sense, ROS can act as chemical signals to specifically induce the biochemical production of antioxidant molecules, including the enzymes of the antioxidant cellular response to stress [36]. Therefore, the use of oxidative stress applied to microalgal cultures can be one of the strategies to achieve increased productivities of those specific target molecules.

In microalgal cultures, oxidative stress can be induced by a range of strategies which involve the use of chemicals. The knowledge of the chemical properties and reactivity of the main added-value molecules obtained from microalgae, as well as the principal metabolic steps of their biosynthesis have allowed to carry out novel research aimed at searching for chemical triggers that enhance accumulation of those molecules. The chemical triggers are different in nature and action mechanisms. According to the latter, these chemicals can be classified into several groups [8]: Oxidative stress inducers, metabolic regulators and metabolic precursors. Besides, chemicals can also be used to induce changes in the physicochemical conditions around the cell environment with the result of the intracellular accumulation of valuable molecules. For instance, surfactant addition to growing microalgal cultures results in foam production containing growing microalgal cells with shifted availability of carbon, oxygen and light [37]. This shifted environment induces physiological responses, including the shifted biochemical profile of valuable microalgal major biomolecules, as further discussed.

In a recent study, Franz et al. [38] described a screening of 42 chemicals for their roles on lipid metabolism in microalgae, and identified 12 chemicals that are capable of enhancing intracellular lipid levels by >100%. Three of these chemicals (epigallocatechin gallate, CDK2 inhibitor 2 and cycloheximide) enhanced intracellular lipids –PUFA included- by 200–400% based on Nile Red fluorescence intensity measurements. In addition, the researchers took a further step to verify these chemicals effectiveness in large-scale cultures and concluded that propyl gallate and butylated hydroxyanisole could be used in large-scale applications considering the low cost of the chemicals and the lipid content increases [38], demonstrating that the application of chemical enhancers could be a valuable, practical approach in addressing the enhanced productivity of microalgae-based products. The main algal molecules with both anti-inflammatory and antioxidant activity, the main chemicals triggering their accumulation and the induction mechanisms in the algal metabolism are summarized in Table 2.

### 2.2. Induced-Oxidative Stress: A Key Strategy to Trigger Accumulation of Anti-Inflammatory Compounds

An increasing interest exists about how microalgae can cope with oxidative stress. Such interest is related to the microalgal potential for the large scale production of valuable molecules accumulated under oxidative stress, the so-called antioxidants. The primary microalgal antioxidant response to oxidative stress conditions imposed to their cultures consists of producing a range of enzymes, such as superoxide dismutase, ascorbate peroxidase, catalase, glutathione reductase and peroxidase, as well as other molecules, such as phytochelatins, pigments, polysaccharides, and polyphenols [61]. In addition, PUFA can also accumulate in response to oxidative stress as described below in this manuscript. As explained, polysaccharides, polyphenols, several pigments—particularly the oxygenated carotenoids called xantophylls—and some PUFA have been demonstrated to display anti-inflammatory activity. Thus, the production of such anti-inflammatory bioactive compounds could in theory be enhanced through chemically-induced oxidative stress of the microalgal cultures (Table 2). As well described by Cirulis et al. [61], chemically-induced production of antioxidant compounds from microalgae requires to research: (i) What chemicals induce oxidative stress in microalgae, (ii) which are the main microalgal metabolism responses to the imposed oxidative stress and what target molecules are produced most abundantly to dissipate the oxidative state, (iii) how to modulate the use of oxidative stress chemical triggers in order to simultaneously boost the accumulation of target molecules whilst obtaining high volumetric productivities, and (iv) how to integrate the acquired knowledge into production processes of target molecules both at laboratory and at large scale.

**Figure 1:**
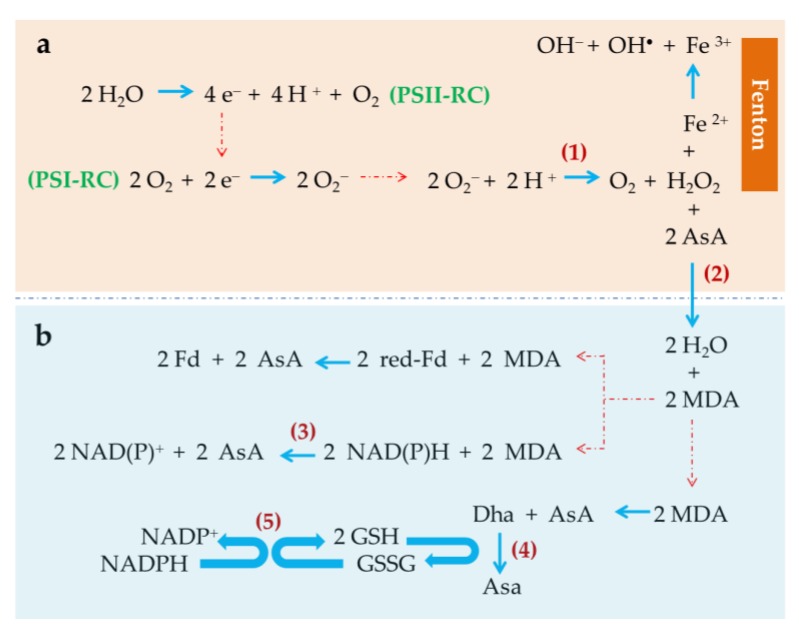
Reactive oxygen species (ROS) production and scavenging mechanisms in photosynthetic organisms. (**a**) Reactive oxygen species production mechanisms, and detoxification of hydrogen peroxide catalyzed by ascorbate peroxidase (2); (**b**) AsA recovery reactions: Enzyme-catalyzed (NAD(P)H-dependent) (3) and spontaneous (red-Fd dependent) biochemical mechanisms of monodehydroascorbate (MDA) reduction, spontaneous disproportion of MDA to Dha and AsA, and enzyme-catalyzed (4) biochemical mechanism of NADPH-GSSG dependent AsA recovery. PSII-RC, photosystem II reaction center; PSI-RC, photosystem I reaction center; AsA, reduced ascorbate; MDA, monodehydroascorbate; red-Fd, reduced ferredoxin; Dha, dehydroascorbate; GSSG, oxidized glutathione; GSH, reduced glutathione; (1) Superoxide dismutase; (2) Ascorbate peroxidase; (3) MDA reductase; (4) Dha reductase; (5) Glutathione reductase.

The simple fact that life occurs in an oxygenated atmosphere results in the continuous presence of oxygen in living cells. As a reactive molecule, oxygen takes part in many chemical and biochemical reactions by means of which ROS permanently arise. ROS are chemically reactive species containing oxygen. ROS include species with unpaired electrons and non-radical species, e.g., hydrogen peroxide, and form during oxygen metabolism through several well identified enzymatic pathways. DNA, proteins and membrane lipids, among other biomolecules, are direct targets of ROS which can alter the normal biological functions of those principal biomolecules: This is the so-called oxidative stress. In photosynthetic cells like those of unicellular microalgae and cyanobacteria, ROS are produced in plastids, mitochondria and cytosol, among other organelles [62]. It is apparent that the light-dependent oxygen production largely contributes to an increase in ROS production in photosynthetic membranes: The greater the photosynthetic activity, the greater the oxygen production in the chloroplasts and, consequently, the potential oxidative damage produced by the ROS generated. Therefore, the reaction centers of photosystem II (PSII) and photosystem I (PSI) play a key role in the production of ROS (Figure 1). In the 1950s Mehler [63] unveiled and described the light-dependent reduction of oxygen to hydrogen peroxide in PSI, therefore superoxide anion being identified as primary reactive oxygen species generated in the chloroplasts [64]. Figure 1 shows details of the chloroplastic ROS-generating reactions (upper frame, a). As indicated, part of the electron flow derived from the water splitting reactions in PSII is derived to the chloroplastic photoreduction of oxygen.

In order to minimize the ROS damage to key cellular processes and functionalities, the cells express diverse antioxidant biochemical mechanisms to neutralize, or at least minimize, the harmful effects of ROS (reviewed by Deawal et al. [18]). The primary antioxidant mechanisms in most organisms include molecules with antioxidant capacity, such as α-tocopherol (vitamin E), glutathione and ascorbic acid, and enzymes with antioxidant activity, such as superoxide dismutase, catalase, ascorbate peroxidase and glutathione peroxidase (Figure 1). In photosynthetic cells other molecules, such as carotenoids, PUFA and polysaccharides also contribute to cope with a harmful excess ROS. When ROS production exceeds the antioxidant capacity of the cells, harmful effects arise. The above referred antioxidant mechanisms contribute to keeping the intracellular physiological contents of ROS at low levels. In this sense, the enzyme superoxide dismutase catalyzes the subsequent disproportion of superoxide anion to oxygen and hydrogen peroxide [64], thus eliminating the oxidative risk associated with the superoxide anion but still generating a highly reactive, toxic compound, hydrogen peroxide. The main detoxification reaction for hydrogen peroxide is its enzyme-mediated reduction to water catalyzed by ascorbate peroxidase (APX). The subsequent group of reactions shown in Figure 1b expresses the reduced ascorbate (AsA) recovery reactions dependent of NAD(P)H or reduced ferredoxin (red-Fd).

The AsA-dependent enzyme-catalyzed reduction of hydrogen peroxide to water is a crucial step to produce an efficient detoxification of ROS. As shown in Figure 1 (frame a), the highly reactive hydrogen peroxide generated in the reduction of superoxide anion by the enzyme superoxide dismutase, can react with chemical species able to reduce it resulting in the production of additional, extremely active oxygen species. This is the case for Fe (II) which reduces hydrogen peroxide resulting in Fe (III), hydroxyl group and hydroxyl free radical production. This is the so-called Fenton reaction. Therefore, in presence of Fe (II), part of the hydrogen peroxide produced in photosynthetic cells can be diverted to ROS generation instead of being detoxified by the action of APX. Accordingly, Fe (II) can trigger oxidative stress in photosynthetic cells when added to a culture medium at optimized concentrations [36,39,44].

Some reports have been published which provide evidence of direct involvement of ROS in the enhanced biosynthesis of microalgal molecules with both antioxidant and anti-inflammatory properties, namely carotenoids and PUFAs. In 1994, Asada [36] described mechanisms of production and action of reactive oxygen species in photosynthetic cells, suggesting that such ROS might be acting as signal molecules to trigger the biosynthesis of a number of antioxidant molecules, carotenoids among them. Further studies have contributed to support the ROS function as signaling molecules and their action as signal transduction processes activators in response to stress. However, the knowledge on the chemical reactions involved in ROS production, their relative rates and the molecular mechanisms by which photosynthetic cells sense ROS is yet scarce. Particularly, for the activation of signaling cell events to happen, ROS with signaling functions (H_2_O_2_, OH^∙^, O_2_) must interact with specific molecular targets. The mechanisms of such interactions and whether each ROS is specifically recognized by a given mechanism or receptors remain unknown [62].

**Figure 2:**
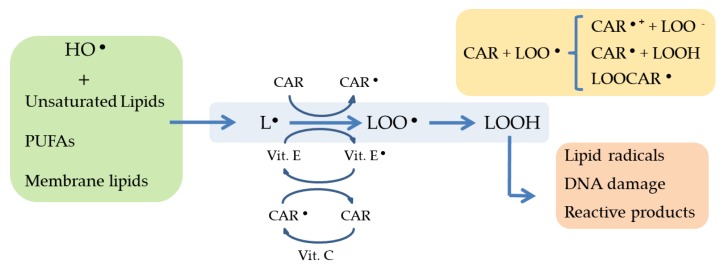
Joint action of ROS scavenging activity exerted by the microalgal anti-inflammatory molecules PUFA and carotenoids. PUFA (unsaturated lipids) and carotenoids are involved in scavenging reactive oxygen species (green frame, left; yellow frame, right); the resulting oxidized peroxidation products are chemically damaging for lipids and DNA, among other molecules (orange frame, right). CAR: Carotenoids; L: Lipid; LOO^●^: Peroxidized lipid; Vit C: Vitamin C; Vit E: Vitamin E.

The role of antioxidants in inflammation has been recently reviewed by Arulselvan et al. [65]. In this respect, carotenoids, PUFA, polysaccharides and other natural molecules have been reported to exert antioxidant properties which may play a key role in the anti-inflammatory response [7,65]. Such antioxidant activity consists of scavenging ROS, and/or dissipating photons excess which cannot be photochemically quenched. The basic principle by which the anti-inflammatory microalgal unsaturated fatty acids and terpenoids (carotenoids, xantophylls) act as ROS scavengers is shown in Figure 2. Whether, and how, the biosynthesis of these antioxidant molecules is triggered by the action of ROS acting as signal transduction process activators remains unclear yet; however, many evidence have been published which prove enhanced biosynthesis and accumulation of those anti-inflammatory antioxidant molecules upon induced oxidative stress in microalgae. Consequently, the addition of certain chemicals which act as oxidative stress inducers should be expected to result in enhanced accumulation of carotenoids, PUFAs and/or polysaccharides and is indeed one of the strategies preferred in recent years [8].

The nature of chemicals that can induce oxidative stress in photosynthetic cells is diverse and the number of those can be enormous. Those being most commonly used in laboratory experiments currently are listed in Table 2 and include hydrogen peroxide, sodium hypochlorite, Fe (II and III), Cu (II), sodium chloride, selenite or selenate, herbicides, such as methyl viologen (MV), salicylic acid (SA), methyl jasmonate (MJ) and giberellic acid (GA), among other compounds. Some of them, for instance SA, MJ and GA, play roles in regulating expression of genes involved in the biosynthesis of anti-inflammatory xantophylls [8,66] and, more interestingly, they can play roles as oxidative stress inducers when used in concentrations that are relatively high in relation to the antioxidant capacity of the microalgal cells. Therefore, some of these compounds which are phytohormones and analogous molecules can trigger the accumulation of anti-inflammatory molecules by either playing regulatory roles in their biosynthetic pathways or directly acting as oxidative stress inducers when added in excess to the microalgal cultures [8]. Moreover, the cell response is mostly proportional to the concentration of the chemical trigger used which allows design production processes based on the modulate action of such a trigger compound on the algal metabolism. A productive process should be that one in which the target molecule accumulation is enhanced whilst growth rates remain sufficiently high as to keep overall productivity also high. From the practical point of view, the selection of an oxidative stress chemical inducer could be made according to at least the following criteria: (a) The chemical should induce modulate responses in algal growth and target molecules biosynthesis rates that are proportional to the inducer concentration, so that the productivity of target antioxidants can also be maximized accordingly; (b) the chemical should not be toxic to photosynthetic growth at low concentrations; (c) the chemical should be compatible with further biomass processing and with specific applications of the target molecules.

In addition to the latter, other chemical strategies producing oxidative stress and addressing the accumulation of microalgal molecules with anti-inflammatory activity are commonly based on nutrient limitation or starvation. It should be noticed that nutrient starvation, particularly inorganic nitrogen, is an effective chemical tool to induce accumulation of lipids—including carotenes and xantophylls—and polysaccharides in microalgae. This subject has been widely reviewed [3,67]. In the following subsections, the most relevant strategies to address the chemically-induced accumulation of anti-inflammatory microalgal compounds are discussed.

### 2.3. Production of Pigments with Anti-Inflammatory Activity

Carotenoids have been found to positively impact the anti-inflammatory cellular response mechanisms and the immunoresponse modulation [7,68,69]. In addition, their antioxidant nature confers carotenoid-enriched diets properties to diminish the risk of suffering from degenerative diseases [70] and protect the eye macula from adverse photochemical reactions [71]. In people over the age of 65, visual sensitivity and vision loss directly has been found to be associated with lutein and zeaxanthin concentrations in the retina [15,72,73]. Astaxanthin, a powerful, natural antioxidant xanthophyll produced by the microalga *Haematococcus pluvialis*, has been proven effective as an anti-inflammatory [7,74,75,76,77]. In addition, astaxanthin was shown to be effective against benign prostatic hyperplasia and against prostatic cancer [70,78]. The protective action of astaxanthin involves an antioxidant mechanism based on the activation of its hydroxyl groups, which results in the formation of an ortho-dihydroxyconjugate polyene system. This polyene system acts as a chain-breaking antioxidant [79].

In photosynthetic organisms, carotenoids may act as accessory pigments in light harvesting functions during the light phase of photosynthesis and may also exert protection of the photosynthetic machinery from excess light and from other oxidative stress chemical factors (e.g., oxidative species, salt, and nutrient deficiency), by scavenging reactive oxygen species (ROS) [3,15,79]. In general, the level of carotenoids in microalgae increases with oxidative stress. Though chlorophyll is also a valuable antioxidant which has been described to display anti-inflammatory properties [80], its content in photosynthetic cells under oxidative stress usually decreases. Thus, the chlorophyll production is by far more efficient in microalgal cultures growing under non-limiting accumulation conditions, in highly dense cultures produced in controlled photobioreactors [81].

Only a limited number of microalgae genera are commonly used in research to test pigment accumulation under oxidative stress, such as *Haematococcus* for astaxanthin, *Chlorella*, *Scenedesmus* and *Botryococcus* for lutein, and *Dunaliella* for β-carotene [23]. A few reports demonstrate that direct addition of hydrogen peroxide triggered astaxanthin accumulation, for example in *Haematococcus pluvialis* [44], *Chlorococcum* [41] and *Chlorella zofingiensis* [47], showing maximal increased astaxanthin contents up to 30% higher than those of standard cultures. Astaxanthin accumulation can also be boosted indirectly, by intracellular production of hydrogen peroxide through oxidative stress induced by addition of selenite to the culture medium. Selenite-mediated oxidative stress induction was unveiled by significantly increased levels of the hydrogen peroxide antioxidant enzyme activity content of the microalgal cells upon selenite addition, which indirectly resulted in increased astaxanthin accumulation in *H. pluvialis* as part of the cellular antioxidant response [40]. Thus, the application of a suitable type and dosage of a given ROS can be potentially used as an efficient chemical tool for large-scale production of anti-inflammatory xanthophylls, such as astaxanthin.

The increased intracellular content of ROS that is required to boost astaxanthin accumulation can also be achieved by the addition of herbicides to microalgal cultures. Herbicides, such as methyl viologen, are commonly used in laboratory research and have been reported to enhance accumulation of astaxanthin in *Haematococcus* [44] and *Chlorococcum* [41] though it is a detriment to the microalgal growth and, obviously, to the quality of the microalgal products. Looking at the large-scale production process feasibility, the use of herbicides implies the associated problem of its toxicity and thus would not be suitable for industrial production. Interestingly, Ma and Chen [41] demonstrated that the hydroxyl (OH) radical might be a key ROS in the molecular signaling pathway that activates the astaxanthin biosynthesis, therefore suggesting a direct effect of a specific ROS on the astaxanthin production pathway induction. Indeed, an early study of Kobayashi et al. [39] demonstrated that several oxidant chemical species, including superoxide anion radical (O_2_^−^), hydrogen peroxide and 2,2′-azo-bis(2-amidinopropane)-dihydrochloride for peroxy radical (AO_2_), and Fe (II), were capable of boosting astaxanthin accumulation in *H. pluvialis*. These chemical species might be playing a role as oxidizers in the oxidative enzyme reactions chain leading to astaxanthin biosynthesis [82].

The use of ferrous iron-Fe (II) as astaxanthin biosynthesis inducer is of particular interest. Fe (II) is an essential metal ion required for microalgal growth, therefore included in the culture medium composition. As described by Kobayashi et al. [39], Fe (II) catalyzes the so-called Fenton reaction by which OH radical generates. When added in excess, Fe (II) addresses the boosted generation of OH radical, thus largely increasing the oxidative state of the cells. As discussed above, OH radical triggers the molecular signaling pathway that activates astaxanthin biosynthesis. Thus, Fe (II) can be effectively used to enhance astaxanthin accumulation, as reported for example for *H. pluvialis* [83] and *Chromochloris zofingiensis* [42]. One of the advantages of Fe (II) as astaxanthin biosynthesis inducer is the dose-response of the microalgal growth II to this ion. This allows for the better control of chemical conditions (Fe (II) concentrations) that can simultaneously address astaxanthin accumulation and microalgal growth [84].

Nitrogen limitation or starvation and high salinity levels induce oxidative stress in microalgal cells and also the subsequent cellular antioxidant responses, including enhanced biosynthesis and accumulation of anti-inflammatory carotenes and xanthophylls [85]. Indeed, astaxanthin and other carotenoids displaying anti-inflammatory activity (e.g., lutein, β-carotene) can accumulate in microalgal cells in response to nitrogen limitation or starvation (astaxanthin, [45,46]; β-carotene, [67]; lutein [50]) and high salinity of the culture medium (astaxanthin, [39]; lutein [51]). Nitrogen starvation-based strategies come with the disadvantage of the time-limited cell viability of the microalgal cultures. Thus, the nitrogen starvation-based pigment production consists of a two-phase process, with the first phase of microalgal growth in nutrient replete medium followed by a second phase of incubation in a nitrogen-starved medium.

In spite of lutein being one of the most valuable carotenoids, chemically-induced accumulation of lutein has not been extensively studied in microalgae. Considering the valuable applications of lutein and the increasing market demand of natural sources of this pigment [16], this is with no doubt a challenging research field for algaers. In microalgae, lutein has been shown to accumulate in cultures subjected to oxidative stress. For instance, the lutein content of *Chlorella zoofingiensis* was shown to increase in response to ROS generated by low concentrations of H_2_O_2_ or NaClO [48]. The cultures were grown heterotrophically and the lutein increase accounted just for about 11% with respect to control cultures, which suggests there is still room for lutein production improvement under optimized process conditions. But not only the use of oxidants triggers lutein biosynthesis in microalgae; induction of oxidative stress based on nutrient starvation [50], or increased salinity (NaCl), have been found to induce increased accumulation of lutein in *C. onubensis*. Interestingly, lutein accumulates at a high intracellular concentration in Fe (II) or Cu (II)-added cultures of *C. onubensis*, an extremophilic microalgal species isolated from the highly acidic environment of Río Tinto (Southwestern Spain) [49]. The large lutein accumulation is probably one of the physiological microalgal responses to the oxidative conditions of acidic habitats which contain relatively high levels of solved metal ions, such as Fe (II), Mn (II), Al (III) and Zn (II) [24].

Almost no studies have been published dealing with ROS-mediated induction of β-carotene biosynthesis, probably due to the efficient chemical strategies applied for β-carotene mass production at industrial scale and the high intracellular β-carotene content and productivities achieved by the only β-carotene producing microalga *Dunaliella salina* which makes the search for novel chemical triggers unnecessary.

### 2.4. Production of Polyunsaturated Fatty Acids (PUFA) with Anti-Inflammatory Activity

The anti-inflammatory properties of PUFA from microalgae have been widely described and recently reviewed by several authors [7,32]. A large number of scientific papers described the effective therapeutic role of PUFA in a variety of inflammatory pathologies, such as Alzheimer, arthritis and lupus [7,86,87], with a demonstrated activity in chemopreventive functions when used as ω-3 PUFA-rich microalgal oil diet [88].

In photosynthetic cells, PUFA carry out a variety of cellular key functions. Besides the principal roles of PUFA as energy storage molecules, cell membrane components and in the physiology of photosynthetic cells, their antioxidant capacity is one of the cellular defense mechanisms against the harmful presence of ROS. In fact, PUFA show affinity towards ROS generated in algal cells subjected to oxidative stress conditions [89]. Indeed, PUFA have been described to react chemically with ROS (Figure 2), which results in the formation of lipid hydroperoxides [90,91] resulting in an altered profile of lipids. This is of course an inevitable consequence of oxygen-dependent life but is often ignored [33]. Particularly, PUFA autoxidation reaction is mediated by ROS, which results in the formation of lipid hydroperoxides as primary products. The lipid hydroperoxides can be further converted into more oxidized products, such as ketones and malonaldehyde. Other oxidation products are hydroxy alkenals which are formed by peroxidation of 4-hydroxy-2-nonenal (HNE), generated by peroxidation of ω-6 PUFAs.

Thus, together with carotenoids and polysaccharides, PUFA are included among the wide range of antioxidant compounds that are produced by microalgal cells to diminish the harmful consequences derived from oxidative stress [92]. However, such antioxidant role obviously causes the time course-dependent decreased proportion of PUFA with respect to saturated fatty acids, in microalgal cells. In spite of this fact, the production of microalgal PUFA can be improved under suitably controlled oxygen levels [33].

Besides the negative impact of ROS in the growth and high-value compounds accumulation of microalgae, ROS seem to play a relevant role in triggering lipid accumulation in microalgae [93,94] though experimental evidence of ROS-mediated lipid accumulation mechanisms is still lacking [33]. The aforementioned apparent contradiction suggests that lipid accumulation could be induced by suitable intracellular ROS levels which could be achieved by subjecting the microalgal cultures to controlled stress conditions, specifically through controlled oxygen levels. In this respect, bioprocess engineering can help to optimize the oxygen supply to the microalgal cultures. Sun et al. [33] suggest this can be done either by direct oxygen regulation strategies or through new bioreactor designs to improve oxygen supply. Overall, PUFA production can thus be enhanced by keeping oxygen concentration in the photobioreactor at relatively low levels, therefore favoring a ROS production low activity in the cells as a mean to stimulate PUFA accumulation whilst minimizing the excess ROS-dependent lipid peroxidation. As low oxygen concentrations are detrimental to the microalgal growth, the production of PUFA-enriched microalgae can be carried out in a two-step process: The first step of biomass production under normal aeration, followed by the second step of cultivation under low oxygen concentration for enhancing PUFA accumulation. This strategy was used by Qu et al. [54] to produce DHA-enriched biomass of *Schizochytrium* sp. Alternatively, simultaneous growth and PUFA production can be carried out at a constant, average oxygen concentration optimized in order to achieve maximal productivities, as described by Huang et al. [95] for growth and simultaneous accumulation of DHA.

As previously described above, chemical factors other than oxygen and ROS also generate oxidative stress. For example, nutrient limitation or deprivation (-N, -P) and high salinity cause increased intracellular ROS level in microalgae [96,97], thus triggering the expression of antioxidant defenses as carotenoids and lipids in the microalgal cells. If the intracellular ROS levels exceed the antioxidant cellular capacity, oxidative stress originates. Some of the consequences were aforementioned and include decreased PUFA proportion due to their reaction against ROS. For example, a reduced content of PUFA was observed in *Phaeodactylum tricornutum* cultures under nitrogen limitation [98], and a trend between increasing EPA yields and decreasing salinity was found in *Pavlova lutheri* [52], both results indirectly suggesting the intense oxidative state arose from nutrient limitation or increased salinity.

In spite of this negative correlation between accumulation of anti-inflammatory PUFA and nutrient limitation or salinity level, the accumulation of these compounds in microalgae can also be enhanced under nitrogen limitation or relatively high salinity but only if these conditions are optimized and applied to the right time of the stress phase, the accumulation being also species-dependent [99,100]. Although the connection between salinity or nutrient starvation and intracellular antioxidant capacity has been often reported [11,50,101], this does not necessarily imply systematic, continuous intracellular accumulation of antioxidants as they can also be involved in coping with intracellular ROS under intense oxidative stress, thus decreasing their concentration in the microalgal cells. Therefore, as the main conclusion, the oxidative stress-mediated accumulation of antioxidant, anti-inflammatory PUFA in microalgae should be carefully addressed by controlling the oxidative pressure and selecting the right experimental time to harvest the PUFA-enriched microalgal biomass.

Conversely, the reduced oxidative damage of microalgal cells growing in a replete culture medium can explain the highest EPA content displayed under nitrogen repletion conditions by *Nannochloropsis oceanica* (above 30% of total fatty acids cell content) [53], the highest DHA content observed in *Pavlova lutheri* (approximately 30% of total fatty acids cell content) [55] and the increased PUFA content in *Tetraselmis marina* [99]. Both nitrogen and phosphorus repletion conditions promote microalgal growth which implies the more active biosynthesis of cell membranes, and particularly tylakoid membranes in light limited high cell density cultures aimed at improving light capture [102].

### 2.5. Production of Sulphated Polysaccharides (sPS) with Anti-Inflammatory Activity

The anti-inflammatory properties of sPS from microalgae have been recently reviewed [32]. The anti-inflammatory properties of sPS-enriched microalgae have been proven in vivo and in vitro for diverse microalgae, such as species of *Chlorella* and *Phaeodactylum* [103,104]. According to the literature, probably the large majority of cyanobacterial polysaccharide structures contains sulfate groups [105] and thus can be considered to be sPS. However, the lack of knowledge on structural data of cyanobacterial polysaccharides limits the possibilities to establish relationships between their chemical structures and biological properties. In addition to it, the cyanobacterial polysaccharide structures can vary depending on species. Thus, further research on the structure of cyanobacterial polysaccharides is required before their real bioactive potential and applications to human health can be evaluated. The current knowledge on the production, extraction and characterization of cyanobacterial polysaccharides has been recently revised in an outstanding review by Delattre et al. [105].

In cyanobacteria, a large fraction of assimilated carbon can be diverted to the synthesis of polysaccharides [106]. Polysaccharides have the function of serving as carbon and energy reserves in microalgae. In addition, a fraction of the produced polysaccharides can be excreted to cover the cells with a mucilage layer to protect them from harsh environmental conditions and/or predators [105]. Some of these cyanobacterial polysaccharides have also been found to display antioxidant activity in addition to their reported anti-inflammatory activity. For example, antioxidant activity has been found in extracellular polysaccharides from *Rhodella reticulata* [34] and *Porphyridium* [107].

Cyanobacterial polysaccharides are also included among the antioxidant molecules produced by microalgae in response to oxidative stress. Cyanobacteria produce exopolysaccharides, which according to literature are sPS, up to more than 20 g·L^−1^ [108,109] in response to a range of physic-chemical conditions. Nutrient limitation and starvation, particularly lack of N, is the classical strategy to induce cyanobacterial polysaccharides accumulation [105], which most probably derives from the N/C ratio metabolic imbalance rather than from the antioxidant cellular response. N limitation, rather than N starvation, might be a more productive strategy. N limitation can be applied to one-phase production strategy in photobioreactors outdoors, by which N concentration is adjusted to limiting levels only when the cultivation conditions are suitable to boost sPS accumulation. Microalgal growth declines under N-limitation, but still a compromise between sPS intracellular accumulation and growth can be achieved in order to maximize sPS productivity. Limitation or starvation of main nutrients other than N, for instance P and S, can also result in polysaccharide accumulation [110], though their effectiveness depends much on the algal species and cultivation conditions, and it has to be specifically determined and optimized in each case [111,112]. As explained by Delattre et al. [105], almost no studies have been conducted to analyze what nutrient does really determine polysaccharides production and how to simplify the culture medium composition.

As explained above, salinity induces oxidative stress in microalgae. Accordingly, and considering the antioxidant character of microalgal polysaccharides, salinity could act as polysaccharide accumulation inducer in microalgae which could use the produced polysaccharides for protective roles. Indeed, the polysaccharide layer around the cells can limit the diffusion of ions through the cell cover [113]. Such this role might be played by polysaccharides produced by several species of cyanobacteria, for instance *Aphanocapsa*, *Anabaena* or *Synechocystis* [105,114,115], which should mostly be sulphated (sPS) and thus expected to display anti-inflammatory activity [32].

### 2.6. Production of Phenolic Compounds with Anti-Inflammatory Activity

Phenolic compounds are secondary metabolites produced by photosynthetic organisms and have awaked interest in recent years, particularly due to their antioxidant character. Based on such properties, the phenolic compounds are known for their benefits to human health. Specifically, the anti-inflammatory properties of microalgal phenolic compounds have been recently reported [116] and contribute to augment the industrial interest of microalgae as raw material for the generation of novel health products. The phenolic compounds found in microalgae and cyanobacteria and cited in the literature include phenolic acids and flavonoids, such as gallate, chlorogenate, cinnamate, gentisic acid, isoflavones, flavanones, flavonols, and dihydrochalcones [59,60].

The action mechanism of anti-inflammatory compounds is performed by a variety of inhibitory activities of pro-inflammatory mediators and/or gene expression [116]. In inflammation, pro-inflammatory cytokines induce the formation of nitric oxide (NO) by inducible nitric oxide synthase, and compounds that inhibit NO production have anti-inflammatory effects. Phenolic compounds, e.g., flavonoids, are among the molecules displaying inhibitory activities of pro-inflammatory mediators. In this regard, a study of Hämäläinen et al. [117] shows that the anti-inflammatory action of flavonoids is due to the inhibitory activity of lipopolysaccharide-induced NF-κB (nuclear factor kappa-light-chain-enhancer of activated B cells) activation. Anti-inflammatory effect of flavonoids consists of inhibiting NF-κB activation only, along with their inhibitory effect on nitric oxide synthase expression and nitric oxide production in activated macrophages.

Phenolic compounds accumulation in microalgae has been directly related to the antioxidant response mechanisms of the cells to increased intracellular oxidative states. Indeed, the anti-inflammatory activity of phenolic compounds is directly related to their antioxidant activity against ROS. Phenolic compounds have been described as radical scavengers based on their role as donors of hydrogen atoms or electrons, producing stable radical intermediates. They can also inhibit iron-mediated ROS formation to prevent the subsequent oxidative stress [92].

However, only a few studies have been reported so far which deal with the accumulation of phenolic compounds in microalgae as a specific cellular response to ROS generated under oxidative stress conditions, for instance, exposure to metal stress [61]. In this regard, a number of factors, such as nutrient level or presence of metal ions can trigger biosynthesis and accumulation of phenolic compounds in microalgae. As an example, El-Baky et al. [60] reported that intracellular accumulation of polyphenols in *Spirulina* was stimulated by nitrogen, both in inorganic (NaNO_3_) or organic (L-phenylalanine) forms. Particularly, the authors reported the presence of phenolic acids and flavonoids predominantly with gallate, chlorogenate, cinnamate and *p*-OH-benzoates. Moreover, in relation to the microalgal antioxidant response, accumulation of phenolic compounds has been described in *Dunaliella tertiolecta* in response to stress produced by addition of Cu (II) or Fe (II). Cu-exposed (790 nmol·L^−1^) *D. tertiolecta* cultures contained 1.4-fold higher intracellular levels of phenolic compounds than standard cultures [59], thus suggesting that the intracellular accumulation of phenolic compounds in microalgae can be triggered by a metal-induced enhanced oxidative state of the microalgal cells.

## 3. Surfactant-Facilitated Accumulation of Anti-Inflammatory Molecules

Microalgae cultivation in foam is a novel concept that consists of growing microalgal cells in surfactant-based foam [118]. A surfactant is a chemical compound that lowers the surface tension between two liquids, or a liquid and a solid, or a gas and a liquid. The chemical structure of a surfactant is composed of zones of different polarity, typically both a polar and a non-polar part. When mixed in culture medium at a given concentration (at least the so-called CMC, critical micelle concentration) and bubbled with air, foam arises. If the culture medium contains growing microalgal cells, microalgae-enriched foam with actively growing cells is produced. In doing so, microalgae can be continuously grown in foam for which specific foam-bed reactors should be designed [118].

The foam-bed photobioreactor is a novel bioengineering concept originally emerged at Wageningen University [119]. In a foam-bed photobioreactor the microalgal cells grow in liquid foams [118], and it has several advantages when compared to liquid cultures: Mass transfer increase, reduction in aeration and biomass harvesting costs, and minimization of water consumption. The successful performance of a foam-bead photobioreactor depends on several factors that have been recently studied [37,120]. Poloxamers, non-ionic surfactants, were demonstrated to be effective for microalgal growth in foam [37].

**Figure 3:**
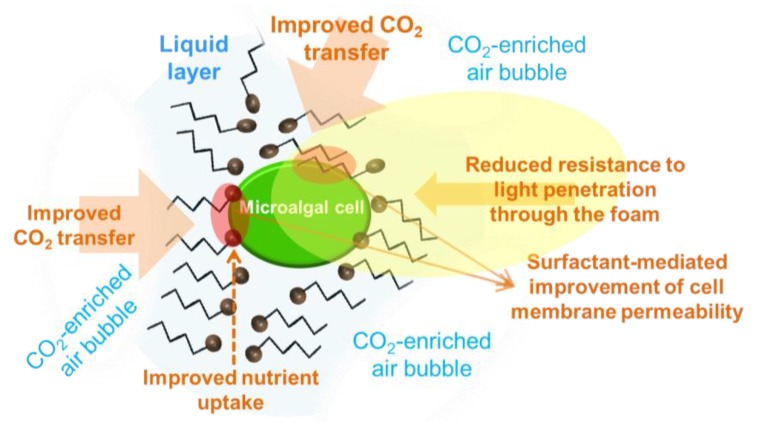
Scheme of physicochemical features that might contribute to enhanced growth and/or shifted biochemical composition of microalgae growing in liquid foams. Symbols: Black sphere with tail, surfactant molecules.

In the foam formed, the microalgal cells grow in the liquid layer that surrounds foam bubbles (Figure 3). The foam is formed by the air volume contained in micelles of surfactant molecules. The non-polar zone of a surfactant molecule is oriented towards the air inside the surfactant micelles whilst the polar zone points to the water layer around the bubble. The cell cover of microalgal cells usually presents electrical charge density which may change towards neutral values during growth. Accordingly, electrostatic interactions between either polar or non-polar zones of the microalgal cell cover and polar or non-polar zones of the surfactant molecules, respectively, may produce molecular disorders in the cell wall and thus influence their functions, including nutrient uptake. This can impact primary metabolism and growth of microalgal cells in foam [1,121], and can result in a modified biochemical composition of the cell. Consequently, cultivation in foam could be investigated as a tool to modify the major biochemical composition of the microalgal cells, i.e., total content of lipids and/or carbohydrates, which include molecules with anti-inflammatory activity as described above in this review.

In a foam-bed photobioreactor, once foam reaches the top part of the cultivation vessel it has to be continuously broken to release the gas contained in the foam bubbles which can produce growth inhibition by oxygen, or lowered growth by carbon limitation. Foam can be broken at the top of the reactor by means of different procedures [118]. Thus, the gas is rapidly released to the atmosphere when foam breaks. This allows for a better control of the oxygen level in the reactor, which can be used to create conditions that stimulate accumulation of high-value compounds, for instance, anti-inflammatory PUFA. Indeed, oxygen concentration has been found to greatly influence the accumulation of value compounds in microalgae [122]. The accumulation of anti-inflammatory compounds in microalgae, such as specific PUFA and carotenoids, can be induced under low oxygen supply. Accordingly, bioprocess engineering strategies are required which lead to an oxygen supply adjusted to the specific demand, and efficient in the oxygen transfer from the gas phase to the culture broth particularly in heterotrophic production [123]. In this regard, novel cultivation strategies and reactor designs that improve mass transfer to the growing cells could help increase productivity of biomass and of the aforementioned target compounds. As described by Janoska et al. [118], the foam-bed photobioreactor has the advantage of high gas holdup which results in both increased mass transfer and lower pressure drop. The increased mass transfer results in higher both carbon and oxygen transfer rates from the gas bubbles to the microalgae cells that grow in the liquid layers between gas bubbles (Figure 3). The improved transfer rates in foam result in increased biomass productivity in well oxygenated microalgal cultures. The opposite, under low oxygen supply, the microalgal metabolism rapidly adapts to enhance PUFA biosynthesis, which can be due to the higher activity of the polyketide synthase (PKS) pathway as it does not require oxygen [124]. PKSs related to PUFA synthase are involved in the biosynthesis of long-chain polyhydroxy alcohols and contribute to the formation of the glycolipid envelope in cyanobacterial heterocysts [125].

The chemical interaction between the polar groups of surfactant molecules and the microalgal cell wall obviously results in a reduced charge density of the microalgal cell cover. In the case of positively charged groups in the surfactant structure, it has been speculated that their neutralization at the cell surface by negatively charged groups should be a reason for better surfactant adsorption to the algal cells [126]. Accordingly, the hydrophobic nature of the oxygen molecule should facilitate its diffusion to the theoretically less polar microalgal cell. Consequently, it could be suggested that the impact of either high or low oxygen concentrations in microalgal cells growing in foam should most probably be faster and more intense than in liquid cultures. This reasoning can partly explain results recently obtained which show that microalgae growing in foam are apparently more sensitive to stress than those growing in liquid cultures. This could be useful as a tool to address the faster accumulation of carbohydrate and lipids, the two major groups of biomolecules with anti-inflammatory activity, in microalgae growing in foam through suitable control of the oxygen and CO_2_ levels in the gas supplied to the cultures, among other factors.

## 4. Conclusions

Besides being used as feed and food, microalgae are currently recognized as natural producers of highly valuable bioactive compounds. Several biomolecules produced by microalgae, namely carotenoids, polyunsaturated fatty acids, sulphated polysaccharides and phenolic compounds, have been found to display anti-inflammatory activity. This finding expands the range of applications of microalgal biomass from healthy diet ingredient in food to nutraceuticals that contribute to diminish the intensity of specific symptoms and/or to prevent specific pathologies related to inflammation processes. Many of those anti-inflammatory biomolecules have also high antioxidant capacity that has been reported to impact human health positively, in particular at advanced ages. Consequently, microalgae are considered to play a relevant role in the future map of novel strategies for the prevention and treatment of inflammatory diseases that includes the use of natural products obtained through well controlled biotechnological processes. The number of microalgae species still to be isolated from nature that could be promising for the accumulation of anti-inflammatory compounds is enormous. To achieve high productivities of target compounds, the isolation of fast-growth species and the enhancement of metabolic fluxes leading to increased biosynthesis of those target products are key aspects. In this sense, chemical tools such as specific triggers of target products and cell growth in foam, are being developed which will be very promising in the short term in order to exploit the production of microalgae enriched in molecules displaying anti-inflammatory activity.

## Figures and Tables

**Table 1:** Biochemical molecules groups of microalgae known to display anti-inflammatory activity.

Biochemical Group	Microalgae	References
Carotenoids	*Haematococcus pluvialis* *Dunaliella salina* *Chlorella sorokiniana* *Synechocystis* sp.	[9,10]
PUFA	*Phaeodactylum tricornutum* *Nannochloropsis gaditana*	[11]
Carbohydrates	*Chlorella vulgaris* *Phaeodactylum tricornutum* *Porphyridium* sp. *Tetraselmis suecica*	[12,13,14]

**Table 2:** Chemical triggers for the production of anti-inflammatory molecules from microalgae.

Anti-Inflammatory Molecule	Chemical Trigger	Induction Mechanism	Microalgae	References
Astaxanthin	H_2_O_2_, SeO_3_^2^, Fe (II)	Oxidative stress	*Haematococcus pluvialis*	[39,40]
	MV	Oxidative stress	*Chlorococcum* sp.	[41]
	Fe (II)	Oxidative stress	*Cromochloris zofingiensis*	[42]
	Jasm., salic. acid	Oxidative stress	*Haematococcus pluvialis*	[43]
	N starvation, NaCl	Oxidative stress	*Haematococcus pluvialis*	[44,45,46]
Lutein	H_2_O_2_, NaClO	Oxidative stress	*Chlorella zoofingiensis*	[47,48]
	Fe (II), Cu (II)	Oxidative stress	*Coccomyxa onubensis*	[3,24,49]
	N starvation	Oxidative stress	*Coccomyxa onubensis*	[50]
	NaCl	Oxidative stress	*Botryococcus braunii*	[51]
EPA	Low oxygen	PUFA stimulation	*Pavlova lutheri*	[52]
	N, P repletion	PUFA stimulation	*Nannochloropsis oceanica*	[53]
DHA	Low oxygen	PUFA stimulation	*Schizochytrium* sp.	[54]
	N, P repletion	PUFA stimulation	*Pavlova lutheri*	[55]
Sulphated polysaccharides	N starvation	Oxidative stress	*Rhodella violacea*	[56]
	P starvation	Oxidative stress	*Phaeodactylum tricornutum*	[57]
	NaCl	Oxidative stress	*Dunaliella salina*	[58]
Phenolic compounds	Cu (II), Fe (II)	Oxidative stress	*Dunaliella tertiolecta*	[59]
	N repletion + Phe	Phenylpr. synth.	*Spirulina platensis*	[60]

Phe: Phenylalanine; Phenylpr. synth.: Phenylpropanoid synthesis stimulation; Jasm., salic. acid: Jasmonate, salicylic acid.
